# Metabotropic Glutamate Receptors Type 3 and 5 Feature the “NeuroTransmitter-type” of Glioblastoma: A Bioinformatic Approach

**DOI:** 10.2174/1570159X22666240320112926

**Published:** 2024-03-20

**Authors:** Matteo Caridi, Marika Alborghetti, Valeria Pellicelli, Rosamaria Orlando, Francesco Ernesto Pontieri, Giuseppe Battaglia, Antonietta Arcella

**Affiliations:** 1 Division of Hematology and Clinical Immunology, Department of Medicine, University of Perugia, Perugia, Italy;; 2 Department of Neuroscience, Mental Health and Sensory Organs, Sapienza University of Rome, Rome, Italy;; 3 Internal Medicine, Sapienza University of Rome, Rome, Italy;; 4 Department of Physiology and Pharmacology, University Sapienza of Roma, Rome, Italy;; 5 IRCCS Neuromed, Pozzilli, Italy;; 6 IRCCS Fondazione Santa Lucia, Rome, Italy

**Keywords:** Glioma, glioblastoma multiforme, metabotropic glutamate receptors type 3 and type 5, glutamate, neurotransmitter-GBM, immunological signature, metabolic signature

## Abstract

**Background:**

Glioblastoma (GBM) represents an aggressive and common tumor of the central nervous system. The prognosis of GBM is poor, and despite a refined genetic and molecular characterization, pharmacological treatment is largely suboptimal.

**Objective:**

Contribute to defining a therapeutic line in GBM targeting the mGlu3 receptor in line with the principles of precision medicine.

**Methods:**

Here, we performed a computational analysis focused on the expression of type 3 and 5 metabotropic glutamate receptor subtypes (mGlu3 and mGlu5, respectively) in high- and low-grade gliomas.

**Results:**

The analysis allowed the identification of a particular high-grade glioma type, characterized by a high expression level of both receptor subtypes and by other markers of excitatory and inhibitory neurotransmission. This so-called neurotransmitter-GBM (NT-GBM) also shows a distinct immunological, metabolic, and vascularization gene signature.

**Conclusion:**

Our findings might lay the groundwork for a targeted therapy to be specifically applied to this putative novel type of GBM.

## 1 INTRODUCTION

Glioblastoma multiforme (GBM) accounts for 12-15% of all intracranial tumors and 60-75% of glial tumours [1]. GBM has an incidence of 3-4 cases per 100,000/year, with a peak between 45 and 75 years of age and is more prevalent in males. GBM has a poor prognosis, with an average survival of about 12 months, because of the high proliferation rate and resistance to the current therapy. Glioma stem cells, which likely originate in zones of active neurogenesis of the adult brain, support tumor growth and are highly resistant to both radio- and chemotherapy [2, 3].

According to the 2021 World Health Organization Classification of CNS cancers, gliomas (astrocytomas and oligodendrogliomas) are subdivided into four grades of severity on the basis of their histologic features (mitotic index, absence or presence of nuclear atypia, neovascularization, and necrosis). GBM is a grade-IV astrocytoma characterized by high mitotic activity, nuclear atypia, cell pleomorphism, extensive neovascularization and/or necrosis [1]. The morphological classification of gliomas has been integrated with the introduction of genetic markers, such as the IDH1 gene encoding the enzyme, isocitrate dehydrogenase type-1, co-deletion of chromosomes 1p-19q, and deletion of X chromosome [3-5]. The DNA alkylating agent, temozolomide, is the gold standard drug in the treatment of GBM, but its efficacy is limited and restricted to GBM cells that do not express the resistance enzyme, O6-methylguanine-DNA methyltransferase (MGMT) [6]. The identification of molecular targets, such as the epidermal growth factor and fibroblast growth factor receptors, the membrane tyrosine kinase, MET, vascular endothelial growth factor, the phosphatidylinositol-3-kinase (PI3K) pathway, and cyclin-dependent kinases, paved the way to the application of precision medicine in the treatment of brain gliomas [7, 8]. Metabotropic glutamate (mGlu) receptors fall into this scenario. mGlu receptors are G-protein coupled receptors activated by glutamate, the major excitatory neurotransmitter in the CNS. mGlu receptors form a family of eight subtypes, subdivided into three groups on the basis of their amino acid sequence and G-protein coupling. Group I includes mGlu1 and mGlu5 receptors, which are coupled to G_q/11_. Their activation stimulates the hydrolysis of phosphatidylinositol-4,5-bisphosphate, with the ensuing formation of the second messengers, inositol-1,4,5-trisphosphate (InsP_3_) and diacylglycerol (DAG). InsP_3_ releases Ca^2+^ from intracellular stores, whereas DAG facilitates the activation of protein kinase C. Group-II (mGlu2 and -3) and group III (mGlu4, -6, -7, and -8) subtypes are coupled to G_i/o_, and their activation inhibits adenylyl cyclase activity [9, 10]. mGlu receptors modulate excitatory synaptic transmission and are found in all elements of the tetrapartite synapse, *i.e*., axon terminals, postsynaptic densities, astrocytes, and microglia (reviewed by Nicoletti *et al.*, 2011) [11]. However, mGlu receptors are also present in cancer cells, where they have been implicated in mechanisms regulating cell proliferation, differentiation, resistance to chemotherapy and oncogenic transformation [12-17]. mGlu receptors have been consistently detected in glioma cells, glioma cell lines, glioma stem cells (GSCs), and bioptic specimens of human gliomas. In rat, C6 glioma cell lines, mGlu receptor ligands displace specifically bound [3H]glutamate with a rank order of affinity that was consistent with the presence of either mGlu1 or mGlu5 receptors [18], and the transcript encoding the mGlu5 receptor was detected in human grade II astrocytoma specimens [19]. mGlu3 and/or mGlu5 receptors were detected in human GBM cell lines [20-22], whe re the two receptors differentially regulate the expression of the glial glutamate transporters, GLAST and GLT-1 [21]. Two studies have shown that glioma cells express mGlu1 receptors and depend on mGlu1 receptor signaling for their viability [23, 24]. Interestingly, mGlu1 receptors are ectopically expressed in melanomas and support tumor spreading in mice [25]. The role of mGu5 receptors in the biology of glioma cells is largely unknown. The evidence that mGlu5 receptor blockade facilitates hypoxic glioma cell death [26] encourages using brain permeant mGlu5 receptor antagonists in experimental animal models of malignant gliomas.

The mGlu3 receptor is the most extensively studied mGlu receptor subtype in glioma cells and is a promising target for therapeutic intervention. The mGlu3 receptor displays a high affinity for glutamate [27] and, therefore, can be potently activated by the glutamate released from glioma cells *via* the glutamate:cysteine antiporter or spread out from the surrounding synapses. The evidence that mGlu3 receptor blockade reduced glioma cell proliferation and restrained tumor growth in mice [22] laid the groundwork for the study of mGlu3 receptors in GSCs. Ciceroni *et al.* (2008) [28] were the first to show that GSCs express mGu3 receptors and that receptor activation sustains the undifferentiated state of GSCs by negatively modulating the action of bone morphogenetic proteins. Pharmacological blockade of mGlu3 receptors with the orthosteric antagonist, LY341495, induced GSC differentiation into astrocytes and 3-month treatment with LY341495 reduced the size of gliomas generated by intracerebral infusion of GSCs in nude mice. In a subsequent study, the same group was able to demonstrate that endogenous activation of mGlu3 receptors increased the resistance of GCSs to temozolomide by enhancing MGMT expression through a signaling pathway that involved PI3K and nuclear factor-κB (NFκB) [29]. Interestingly, mGlu3 receptor antagonists enhanced temozolomide toxicity in cultured GSCs and synergized with temozolomide in reducing the size and spreading of brain tumors originating from GSCs in mice [29]. All these findings have been largely confirmed by most recent studies [30-32], which, together, support the use of mGlu3 receptor antagonists in the treatment of malignant gliomas. The mGlu8 receptor may act as a counterpart of the mGlu3 receptor because glioma cell clones with down-regulated mGlu8 receptors showed a higher proliferation rate and increased resistance to chemotherapy [33].

One of the limitations in the development of new therapeutic targets is the heterogeneity of human gliomas, which may generate a high variability in drug response. Interestingly, patients with surgically removed GBM treated with temozolomide showed a longer overall survival if the resected tumor had low levels of mGlu3 transcript, and the methylation state of the MGMT gene promoter influenced survival only in patients with low mGlu3 receptor mRNA in the tumor [29]. Thus, treatment with mGlu3 receptor antagonists or negative allosteric modulators (NAMs) is expected to be effective in patients with high expression levels of mGlu3 receptors in GBM, who are otherwise resistant to adjuvant therapy with temozolomide. It is important to identify a tumor signature and a possible tumor subtype that predicts the expression levels of mGlu3 receptors in malignant gliomas. We have used a bioinformatics approach extending the analysis to the mGlu5 receptors because mGlu3 and mGlu5 receptors functionally interact in brain tissue [34] and are both expressed in glioma cells.

## 2 MATERIALS AND METHODS

To examine the expression of mGlu3 and mGlu5 receptors in GBM, available datasets (GSE15824, GSE23806, GSE36245 and GSE53733) were obtained from Gene. Expression Omnibus (GEO, https://www.ncbi.nlm.nih.gov/geo/).

GSE15824 included genes from 30 brain tumor samples. GSE23806 included a large panel of human GBM stem-like cell lines corresponding to primary tumors and conventional glioma cell lines, from which we selected 12 primary GBM tumor samples. GSE36245 included 46 GBM samples from patients of different ages. GSE53733 included genes from 70 GBM patients of the German Glioma Network.

The Affymetrix Human Genome U133 Plus 2.0 Array (HG-U133_Plus_2) slide platform was used. Detailed descriptions of the experimental design and procedures can be retrieved from the relative publications [35-40].

Data were analyzed with R studio (RStudio: Integrated Development for R. RStudio, PBC, Boston, MA) using the following packages: “affy,” “impute,” “tidyverse,” “cluster,” “factoextra” and “Limma” [41-46].

Designed probes were analyzed using the “mas5calls” function, which performs a Wilcoxon signed rank-based gene expression presence/absence detection algorithm; only probes with at least 75% of samples defined as present were analyzed (Present or “P” represents a call of present, assuming that data do not represent an absent transcript). Among different probes that identify the same gene, we selected the one with higher variance.

The expression measures were obtained with RMA, a function that converts an AffyBatch into an ExpressionSet using the robust multi-array average (RMA) expression measure.

We only analyzed samples with “*P*-value” for both mGlu3 and mGlu5 receptors.

In order to investigate the differential expression of cluster of genes associated with immune response, hypoxia, vascular response, and cell proliferation, we have applied the same gene panel used by Reifenberger in 2014 for the molecular characterization of long-term survivors of GBM through a genomic and transcriptomic analysis [47].

In order to obtain the missing data, we performed the k-nearest neighbor algorithm (KNN), a machine learning method that allows to forecast of missing values after a training phase of the algorithm (consists of storing the feature vectors and class labels of the training samples). The KNN was performed with the impute.knn function, which imputes missing expression data using the nearest averaging neighbor. The k value (number of neighbors to be used in the imputation) was established as the square root of the number of samples.

To evaluate the GBM hierarchical clustering based on mGlu3 and mGlu5 receptor expression, we determined the optimal number of clusters with the average Silhouette Method; then, starting from the mGlu3 and mGlu5 receptor expression matrix, we computed a dissimilarity matrix and performed an agglomerative hierarchical clustering.

We used a Shapiro-Wilk test as a normality test on the expression data matrix; based on the normality parameter, correlations between genes were obtained by Pearson and Spearman's rank correlation analysis for parametric and non-parametric data, respectively.

Differences in gene expression among groups were obtained using unpaired two-tailed Student’s t-test with Welch's correction (parametric data); non-parametric data were analyzed with the Mann-Whitney test.

Differentially expressed genes and their significance were identified by analyzing the datasets with the R-package “Limma.” In order to avoid false positive data, we have confirmed the data using the “mas5calls” function and analyzing only probes with a present call. We subsequently performed a test with Welch's correction when the data’s distribution was normal; Mann Whitney test was used in non-normal data. Statistical analysis was performed with GraphPad Prism 9.4.1 (GraphPad Software, San Diego, CA, USA) Microsoft Excel 16.16.3.

For comparisons between low-grade glioma and GMB tissues, the expression dataset was obtained by the TCGA GBMLGG dataset (515 low-grade glioma samples and 152 GBM samples) [48] and the Rembrandt dataset (225 low-grade gliomas samples and 219 GBM samples) [49]. Differential expression was assessed using the t-test with Welch's correction when the data’s distribution was normal; Mann Whitney test was used in non-normal data.

In order to validate the results on differentially expressed genes and differential expression of clusters of genes associated with the different signatures, we repeated our analysis using the TCGA GBM dataset (528 GBM samples) as described above [50]. The TCGA database can be downloaded from the GlioVis data portal (http://gliovis.bio-73info.cnio.es/) [51].

For the correlation between mGlu3 and mGlu5 expression and clinical parameters, we used Chi-square analysis. Comparison of Survival Curves was effectuated using the “Log-rank (Mantel-Cox) test.” The results shown here are in whole or part based upon data generated by the TCGA Research Network: https://www.cancer.gov/tcga.

## 3 RESULTS

### 3.1 Hierarchical Cluster Analysis Identified a GBM Group with High Expression of mGlu3 and mGlu5 Receptors

We first examined the expression levels of the genes encoding the mGlu3 and mGlu5 receptors (GRM3 and GRM5, respectively) in GBM samples from 83 patients and in samples of lower-grade gliomas from 15 patients; the overall analysis showed a greater expression of both mGlu3 and mGlu5 receptors in GBM with respect to lower grade gliomas (*p =* 0.0018 and *p =* 0.0221 respectively).

**Fig. (1):**
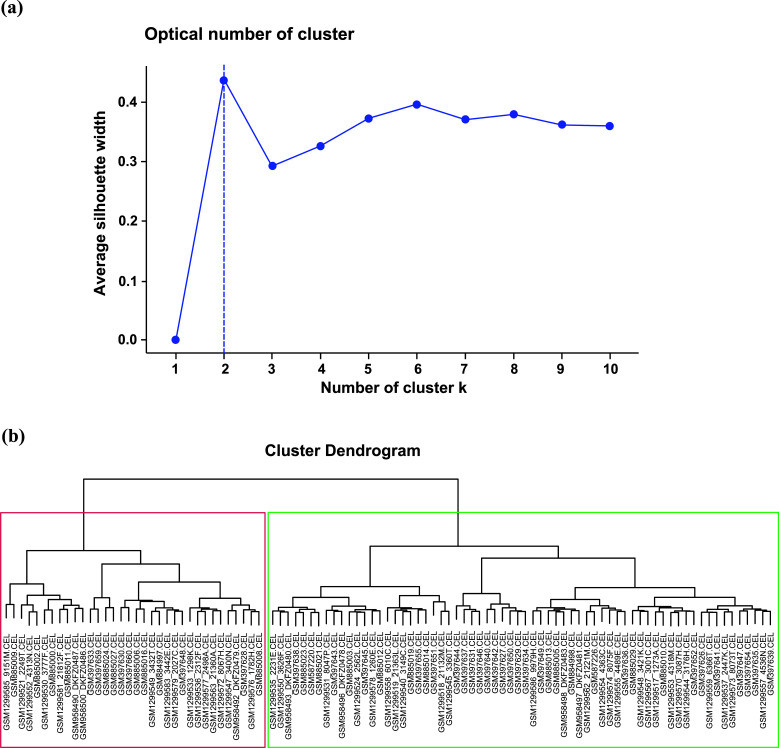
(**a**) the optimal number of clusters determined with the Silhouette method; (**b**) Agglomerative hierarchical clustering of 83 GBM samples identify two main groups of GBM: the NT-GBM in red and the NOS-GBM in green.

We performed a hierarchical cluster analysis of GBM based on mGlu3 and mGlu5 receptor expression; “two” was the optimal number of clusters determined with the Silhouette method (Fig. **1a**). We then performed an agglomerative hierarchical clustering and analyzed the two GBM subgroups (Fig. **1b**). As shown in Fig. (**2**), the first subgroup, named “neurotransmitter (NT)-type,” showed a higher expression of mGlu3 and mGlu5 receptors (*p* < 0.0001) with respect to the second subgroup (the Not Otherwise Specified or “NOS” subgroup).

**Fig. (2):**
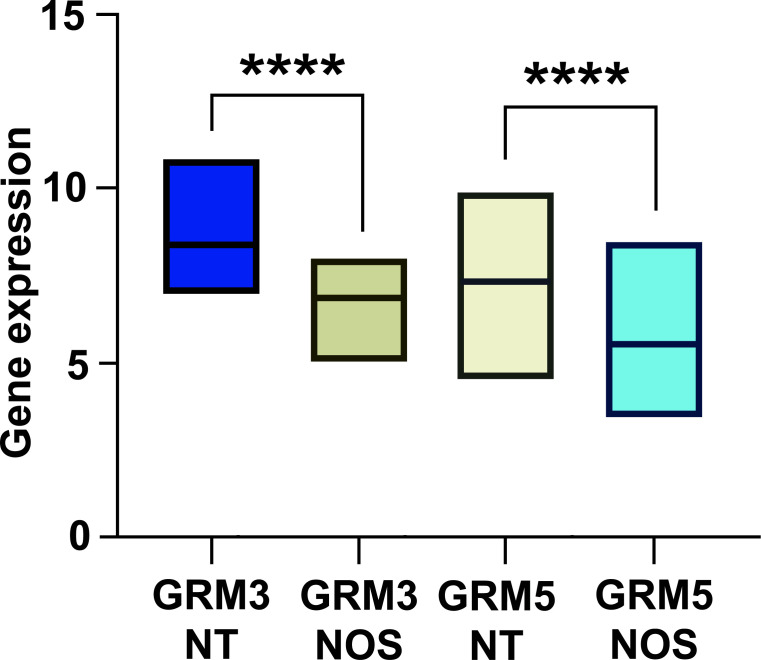
The NT group displays a significantly higher expression of mGlu3 and mGlu5 receptors (****p < 0.0001).

Afterward, we made an individual comparison of the two GBM subgroups with low-grade gliomas, finding that only the NT subgroup showed a higher expression of mGlu3 and mGlu5 receptors (Fig. **2**).

**Fig. (3):**
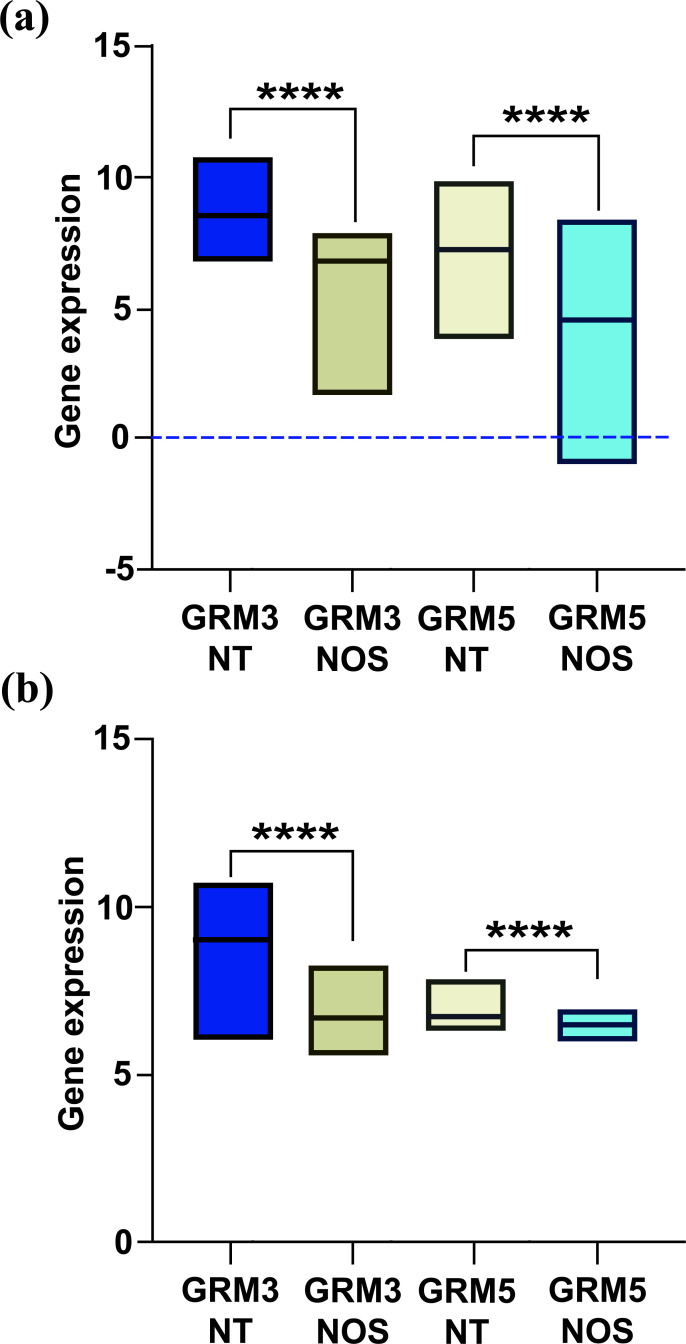
(**a**) the NT-group displays a significantly higher expression of both mGlu3 and mGlu5 receptors than the NOS-group in the TGCA LGGGBM dataset (****p < 0.0001); (**b**) the NT-group displays a significantly higher expression of both mGlu3 and mGlu5 receptors than NOS-group in Rembrandt dataset (*p <* 0.0001).

We used the same approach to cluster the GBM samples from the TCGA and Rembrandt datasets (737 low-grade gliomas and 371 GBM samples). The hierarchical cluster analysis identified two different subgroups in GBM samples, similar to the previous research. These two identified groups differ in the expression levels of both mGlu3 and mGlu5 receptors, where the “NT-type” expresses significantly high levels of the receptors as compared to the “NOS-type” (*p <* 0.0001) (Fig. **3**). The different expression levels of GRM3 and GRM5 in “NT-type” and “NOS-type” was confirmed; however, the difference between low-grade gliomas and GMB was variable and dataset-dependent.

Analyzing microarray datasets, no difference in the IDH1 or H3F3a mutational status was found between the two GBM subgroups, but the value of this analysis is limited by the low number of samples used for IDH1 and H3F3a analysis.

Repeating analysis in the TGCA dataset, no difference in MGMT methylation status, IDH1 mutational status, CpG island methylator phenotype, gender and age were found between the two GBM subgroups, but the analysis is limited by the low number of samples with a strict molecular characterization.

**Fig. (4):**
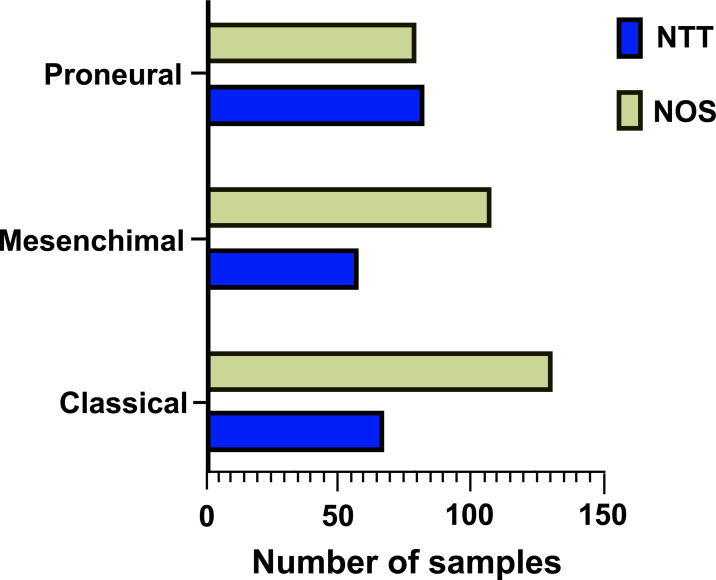
NT type (NTT) is observed more frequently in the proneural GBM subtype rather than classical and mesenchymal ones.

A significantly higher incidence of “NT-type” was observed in proneural subtype GBM rather than classical or mesenchymal ones (Chi-square, df 12,69; *p =* 0.0018) (Fig. **4**).

### 3.2 The NT-type Group Displays Different Immunity, Vascular and Hypoxic Signatures with Respect to the NOS Group

We attempted to identify specific signatures of GBM with a high expression of mGlu3 and mGlu5 receptors, examining a cluster of genes associated with immune response, hypoxia, vascular response/extracellular matrix (ECM), and cell proliferation.

Bioptic samples containing the NT-type GBM subgroup showed:

#### 3.2.1 A Lower Expression of the Following Genes Related to Innate or Adaptive Immunity

FPR1 (encoding the formyl receptor peptide 1; *p <* 0.0045), TLR2 (encoding Toll-like receptor 2; *p =* 0.0263), FCGR2A (encoding the Fc fragment of IgG low affinity IIa receptor; *p =* 0.0051), C1R (encoding the r subcomponent of complement component 1; *p =* 0. 0.0019), C1QB (encoding the β-chain, q subcomponent of complement component 1; *p =* 0.0161); MAFB (encoding the v-maf avian musculoaponeurotic fibrosarcoma oncogene homolog B; *p =* 0.0075); SOCS3 (encoding the suppressor of cytokine signaling 3; *p =* 0.0054); CD44 (encoding for CD44 molecule; *p =* 0.0002); TGFB2 (encoding the transforming growth factor β 2; *p =* 0.0497); CSF1 (encoding the colony stimulating factor 1; *p =* 0.0029); FCGRT (encoding the Fc fragment of IgG receptor transporter α; *p =* 0.0003); TNFAIP3 (encoding the tumor necrosis factor α induced protein 3; *p =* 0.0067); ILR1 (encoding the interleukin 1 receptor type I; *p =* 0.0053); TGFBI (encoding the transforming growth factor β-induced; *p <* 0.0001); SLA (encoding the Src-like-adaptor; *p =* 0.0048); FCGR3A///FCGR3B (encoding the Fc fragment of IgG low affinity IIIa receptor and the Fc fragment of IgG low affinity IIIb receptor; *p =* 0.0348); C1QA (encoding the α chain, q subcomponent of complement component 1; *p =* 0.0021); CAPG (encoding the gelsolin-like, actin filament capping protein; *p =* 0.0033); VCAM (encoding the vascular cell adhesion molecule 1; *p =* 0.0002), (Table **1a**).

#### 3.2.2 A Lower Expression of the Following Genes Related to Hypoxia

LOX (encoding the lysyl oxidase; *p =* 0.0014); VEGFA (encoding the vascular endothelial growth factor A; *p =* 0.0002); IGFBP3 (encoding the insulin-like growth factor binding protein 3; *p =* 0.0001); ADM (encoding the adrenomedullin; *p =* 0.0027); CAV1 (encoding the caveolin 1; *p =* 0.0005); CTSB (encoding the cathepsin B; *p =* 0.0121); PLAUR (encoding the plasminogen activator urokinase receptor; *p =* 0.0092); STC1 (encoding the stanniocalcin 1; *p <* 0.0001); ANGPTL4 (encoding the angiopoietin-like 4; *p =* 0.01); PGK1 (encoding the phosphoglycerate kinase 1; *p =* 0.0002); LDHA (encoding the lactate dehydrogenase A; *p =* 0.009); CA12 (encoding the carbonic anhydrase XII; *p =* 0.0323), (Table **1b**).

#### 3.2.3 A Lower Expression of the Following Genes Related to Extracellular Matrix

THBS1 (encoding the thrombospondin 1; *p =* 0.0592; COL3A1 (encoding the collagen type III α1; *p =* 0.0001); ADAM12 (encoding the ADAM metallopeptidase domain 12; *p =* 0.0236); MGP (encoding the matrix G1a protein; *p =* 0.0095); CDH11 (encoding the cadherin 11 type 2 OB-cadherin; *p =* 0.0004); CD163 (encoding the CD163 molecule; *p =* 0.0032); CYP1B1 (encoding the cytochrome p450 family 1 subfamily B polypeptide 1; *p =* 0.0515); COL6A3 (encoding the collagen type VI α3; *p =* 0.0005); IL1R1 (encoding interleukin 1 receptor type 1; *p =* 0.0053); FN1 (encoding the fibronectin 1; *p =* 0.0006), (Table **1c**).

#### 3.2.4 A Lower Expression of the Following Genes Related to Proliferation

CENPF (encoding the centromere protein F; *p =* 0.0562); TK1 (encoding the soluble thymidine kinase 1; *p =* 0.0121), (Table **1d**).

We repeated the analysis using samples provided by the TGCA GBM dataset; the NT-group showed:

(1) A lower expression of the following genes related to innate or adaptive immunity: C1R (*p =* 0. 0.003); SOCS3 (*p =* 0.0065); CD44 (*p <* 0.0001); CSF1 (*p =* 0.0142); TNFAIP3 (*p =* 0.0225); TGFBI (*p <* 0,0001) (Table **2a**); a higher expression of the following genes: CSF1R (encoding for colony-stimulating factor 1 receptor; *p <* 0.0001); FPR1 (*p =* 0.0343); A2M (encoding for α-2-macroglobulin; *p =* 0.008); CD86 (encoding for CD86 molecule; *p =* 0.0014); AIF1 (encoding for allograft inflammatory factor 1; *p =* 0.0004); C1QA (*p =* 0.0206); SYK (encoding for spleen tyrosine kinase; *p =* 0.0171), (Table **2a**).

(2) A lower expression of the following genes related to hypoxia: VEGFA (*p <* 0.0001); IGFBP3 (*p =* 0.0014); ADM (*p <* 0.0001); CAV1 (*p =* 0.0062); STC1 (*p =* 0. 004); LGALS3 (encoding for lectin, galactoside-binding, soluble 3; *p <* 0.0001); PGK1 (*p <* 0.0001); LDHA (*p <* 0.0001); a higher expression of SPP1 (encoding for secreted phosphoprotein 1; *p* < 0.0001), (Table **2b**);

(3) A lower expression of the following genes related to extracellular matrix: THBS1 (*p =* 0.0006); COL3A1 (*p =* 0.0156); ADAM12 (*p =* 0.0004); CDH11 (*p =* 0.003); COL6A3 (*p =* 0.0007), (Table **2c**).

(4) A lower expression of the following genes related to proliferation: RRM2 (encoding for ribonucleotide reductase M2; *p <* 0.0001); TOP2A (encoding for topoisomerase DNA II α; *p =* 0,0195); UBE2C (encoding for ubiquitin-conjugating enzyme E2C; *p <* 0.0001); TRIO (encoding for trio Rho guanine nucleotide exchange factor; *p =* 0.0132); POLE2 (encoding for polymerase (DNA direct) epsilon 2, accessory subunit; *p <* 0.0001); CHEK1 (encoding for checkpoint kinase 1; *p <* 0.0001); CKS2 (encoding for CDC28 protein kinase regulatory subunit 2; *p =* 0.0037); TK1 (*p <* 0.0001), (Table **2d**).

### 3.3 The NT Group is Characterized by a “Neurotransmitter” Signature and Displays Several Differential Expressed Genes

We extended the analysis to individual genes that are differentially expressed in the two subgroups of GBM and associated with a high or low expression of mGlu3 and mGlu5 receptors. The NT subgroups showed a greater expression of the following genes related to glutamatergic or GABAergic neurotransmission: GABRB2 and GABRB3, encoding the β_2_ and β_3_ subunits of GABA_A_ receptors (*p =* 0.0002 and *p* < 0.0001, respectively); GRIA2, encoding the GluA_2_ subunit of AMPA receptors (*p =* 0.0021); and GRID1, encoding the δ_1_ subunit of ionotropic glutamate receptors subunit (*p =* 0.0007). This supports the neurotransmitter phenotype of the NT GBM subgroup.

Other differentially expressed genes included SCD1 (encoding the enzyme stearoyl-CoA desaturase-1) (*p <* 0.0001) and FAD2H (encoding the enzyme fatty acid 2-hydroxyase) (*p <* 0.0001, which are both involved in fatty acid metabolism, and three myelin- related genes: MOG, encoding the myelin oligodendrocyte glycoprotein (*p =* 0.0003), MOBP, encoding the myelin-associated oligodendrocyte basic protein (*p* < 0.0001), and MBP, encoding the myelin basic protein (*p =* 0.0008).

Finally, we analyzed the correlations between mGlu3 and mGlu5 receptor expression and genes differentially expressed in the two GBM subgroups, finding that most of these genes showed a high correlation with both mGlu3 and mGlu5 receptors (Table **3**).

Similar results were obtained from the analysis of TCGA dataset, resulting less expressed by the “NT-Type” GABRB3 (*p* < 0.0001); GRIA2 (*p <* 0.0001); KCNK10, encoding for potassium channel subfamily K, member 10 (*p =* 0002); SCN2A, encoding for sodium channel voltage-gated type II α subunit (*p <* 0.0001); ANK3, encoding for ankyrin 3 (*p <* 0.0001); FAD2H (*p <* 0.0001); MOG (*p <* 0.0001); MOBP (*p* < 0.0001); and MBP (*p <* 0.0001), (Table **4**).

### 3.4 The NT-type and the NOS-type do not Differ in Terms of Overall Survival

**Fig. (5):**
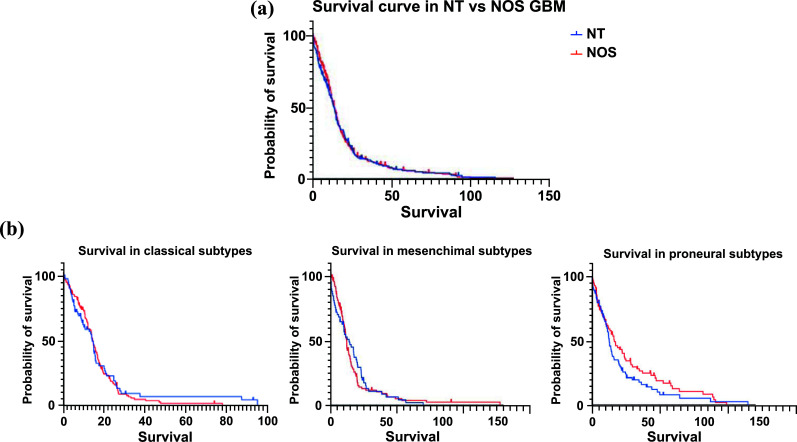
(**a**) No difference in survival between NT-group and NOS-group (*p =* 0,6917); (**b**) NT-group showed the worst prognosis in the proneural subgroup but not in classic and mesenchymal subtypes.

Finally, we tried to clarify whether the different expressions of mGlu3 and mGlu5 receptors in GBM could influence overall survival (OS) and progression-free survival (PFS). Among datasets, the only one that provides accurate information about the OS is the TGCA dataset. The analysis did not reveal significant differences in terms of OS between “NT-group” and “NOS-group” (Fig. **5a**). However, analyzing OS in the different GBM subtypes, we could observe a trend of worst prognosis of “NT-type” in proneural subtypes rather than classic or mesenchymal GBM (Fig. **5b**). More studies are required in order to understand these data, which are consistent with those demonstrated by Ciceroni *et al.* [29].

Accurate information about the PFS was not provided by any of the datasets used for our research.

## 4 DISCUSSION

Our computational analysis suggests that mGlu3 and mGlu5 receptors represent potential molecular markers of GBM. This is in line with the evidence that levels of the transcript encoding the mGlu3 receptors in tumor specimens were inversely related to the survival of patients with grade GBM undergoing surgery and adjuvant treatment with temozolomide [29]. We focused on mGlu3 and mGlu5 receptors because these receptors functionally interact in brain tissue, with mGlu3 receptors boosting mGlu5 receptor signaling [34]. An attractive hypothesis is that this form of receptor cross-talk also occurs in glioma cells and supports cell proliferation and chemoresistance.

As underscored in the Introduction, mGlu5 receptors are coupled to G_q/11_, and their activation leads to the formation of InsP_3_ and DAG, with ensuing intracellular Ca^2+^ release and PKC activation (reviewed by Nicoletti *et al.*, 2011) [11]. These signaling molecules might have a profound impact on basic mechanisms of cancer cell biology, such as proliferation and survival. It will be interesting to examine whether the functional cross-talk between mGlu3 and mGlu5 receptors also exists in GBM cells or GSCs.

Interestingly, mGlu3 and mGlu5 receptors appeared to be highly expressed by a subgroup of GBM characterized by the expression of established markers of glutamatergic and GABAergic neurotransmission, denominated “NT-type” GBM. Only the “NT-type” GMB showed an enhanced mGlu3 and mGlu5 receptor expression with respect to low-grade gliomas, and, therefore, the two receptors cannot be considered as biochemical markers of GBM with respect to other types of gliomas but may facilitate the identification of a specific GBM subgroup. The proposed “NT-type” could not be differentiated on the basis of the mutational state of IDH1 and H3F3a, but the data were biased by the low sample size. It will be interesting to examine the association of mGlu3 and mGlu5 receptors with genetic markers that predict the evolution of low-grade gliomas into GBM, such as chromosome X deletion [1, 8].

Besides genes related to glutamatergic and GABAergic transmission, genes related to innate or adaptive immunity were differentially expressed by the “NT-type” GBM. This suggests that the “NT-GBM” has a different immune signature concerning other gliomas. Several genes typically expressed in macrophages, microglia, and lymphocytes or associated with macrophage activation were differentially expressed in “NT-GBM ” compared with other gliomas. It is possible that owing to the immune-related phenotype, “NT-GBM” exhibits a lower level of immune infiltration and, therefore, a reduced response to immunotherapeutic treatments. This attractive hypothesis warrants further investigation. We have also found that a cluster of genes implicated in hypoxia and angiogenesis was lesser expressed in “NT-GBM” than in “NOS-GBM”. This might result in a less vascularized microenvironment, which might limit cancer cell survival. A cluster of genes related to the extracellular matrix also showed a lower expression in “NT-GBM”. As opposed to what is observed in peripheral tissues, the ECM in CNS lacks collagen, fibrinogen and laminin and is predominantly formed by proteoglycans, hyaluronic acid and tenascin C (reviewed by Vollmann-Zwerenz *et al.*, 2020) [52]. It has been reported that the siRNA-induced downregulation of the chondroitin sulphate proteoglycan, versican V1, reduced the migration of high-grade glioma cells [53]. Therefore, the lower expression of ECM-related genes might restrain the migration capacity of “NT-GBM cells”.

Using samples from the TGCA dataset, a proliferative signature seems to characterize the “NT-GBM” versus the “NOS-GBM.” This difference probably depends on the increase in sample size.

“NT-GBM” was also characterized by a differential expression of genes involved in lipid metabolism, such as SCD1. SCD1, which is heterogeneously expressed in gliomas, protects cancer cells against lipotoxicity by promoting the formation of monounsaturated fatty acids, and its inhibition increases apoptotic cell death and cell vulnerability to chemotherapy [54]. Interestingly, IDH1 mutations cause changes in the expression of SCD1 in glioma cells [55-58]. Other genes differentially expressed by “NT-GBM” include fatty acid 2-hydroxylase and genes encoding proteins that are involved in myelin formation. Although the two subgroups do not differ in terms of OS, the metabolic signature that we have identified in “NT-GBM” may pave the way to targeted therapy for this glioma subtype and consequently affect both OS and PFS.

## CONCLUSION

In conclusion, our computational analyses allowed the identification of a putative novel GBM-type expressing high levels of mGlu3 and mGlu5 receptors, as well as other genes related to glutamatergic and GABAergic neurotransmission. Although the impact of glutamate and GABA receptors on the proliferation, survival, and migration of “NT-GBM” cells remains to be explored, we speculated that a list of some of these receptors might be targeted for therapeutic intervention. Previous studies have demonstrated that the expression of the gene encoding the mGlu3 receptor positively correlates with the severity of high-grade gliomas in humans, and pharmacological blockade of mGlu3 receptors restrains glioma growth has enhanced glioma cells vulnerability to temozolomide in preclinical models [28, 29]. Despite the limitations of our pure bioinformatics approach, our findings may encourage the development of selective mGlu3 receptor antagonists or negative allosteric modulators for the treatment of “NT-GBM” in conformity with the principles of precision medicine.

## Figures and Tables

**Table 1a:** Immune signature - Positive values indicate a higher expression in the NOS group.

**Gene**	**Gene Title**	**Statistical Analysis**	***P* Value**	**Difference between Means**	**Difference: Hodges-Lehmann**
CSF1R	Colony-stimulating factor 1 receptor	Unpaired t-test with Welch's correction	0.2361	0.25	-
**FPR1**	Formyl peptide receptor 1	Unpaired t-test with Welch's correction	0.0045	0.63	-
A2M	Alpha-2-macroglobulin	Unpaired t-test with Welch's correction	0.3297	0.19	-
**TLR2**	Toll-like receptor 2	Unpaired t-test with Welch's correction	0.0263	0.44	-
**FCGR2A**	Fc fragment of IgG, low-affinity IIa, receptor	Unpaired t-test with Welch's correction	0.0051	0.61	-
**C1R**	Complement component 1, r subcomponent	Unpaired t-test with Welch's correction	0.0019	0.93	-
**C1QB**	Complement component 1, q subcomponent, B chain	Unpaired t-test with Welch's correction	0.0161	0.56	-
TGFBR2	Transforming growth factor, beta receptor II	Unpaired t-test with Welch's correction	0.0768	0.42	-
**CTSH**	Cathepsin H	Unpaired t-test with Welch's correction	0.034	0.48	-
**MAFB**	v-maf avian musculoaponeurotic fibrosarcoma oncogene homolog B	Unpaired t-test with Welch's correction	0.0075	0.61	-
**SOCS3**	Suppressor of cytokine signaling 3	Unpaired t-test with Welch's correction	0.0054	1.15	-
**CD44**	CD44 molecule (Indian blood group)	Unpaired t-test with Welch's correction	0.0002	0.69	-
**TGFB2**	Transforming growth factor, beta 2	Unpaired t-test with Welch's correction	0.0497	0.65	-
**CSF1**	Colony-stimulating factor 1 (macrophage)	Unpaired t-test with Welch's correction	0.0029	0.32	-
IL13RA1	Interleukin 13 receptor, alpha 1	Unpaired t-test with Welch's correction	0.2972	0.23	-
**FCGRT**	Fc fragment of IgG, receptor, transporter, alpha	Unpaired t-test with Welch's correction	0.0003	0.37	-
**TNFAIP3**	Tumor necrosis factor, alpha-induced protein 3	Mann Whitney test	0.0067	-	0.48
**IL1R1**	Interleukin 1 receptor, type I	Mann Whitney test	0.0053	-	0.78
**TGFBI**	Transforming growth factor, beta-induced	Mann Whitney test	<0.0001	-	1.37
CXCL9	Chemokine (C-X-C motif) ligand 9	Mann Whitney test	0.1338	-	0.25
CD86	CD86 molecule	Mann Whitney test	0.6633	-	0.08
**SLA**	Src-like-adaptor	Mann Whitney test	0.0048	-	0.41
**FCGR3A ///FCGR3B**	Fc fragment of IgG, low-affinity IIIa, receptor (CD16a) /// Fc fragment of IgG, low-affinity IIIb, receptor (CD16b)	Mann Whitney test	0.0348	-	0.53
AIF1	Allograft Inflammatory Factor 1	Mann Whitney test	0.1152	-	0.23
IL10RA	Interleukin 10 receptor, alpha	Mann Whitney test	0.0759	-	0.29
**C1QA**	Complement component 1, q subcomponent, A chain	Mann Whitney test	0.0021	-	0.70
**CAPG**	Capping protein (actin filament), gelsolin-like	Mann Whitney test	0.0033	-	0.59
SYK	Spleen tyrosine kinase	Mann Whitney test	0.0743	-	0.25
B2M	Beta-2-microglobulin	Mann Whitney test	0.944	-	0.02
**VCAM**	Vascular cell adhesion molecule 1	Mann Whitney test	0.0002	-	1.09

**Note:** Negative values indicate a higher expression in the NTT group.

**Table 1b:** Hypoxia signature - Positive values indicate a higher expression in the NOS group.

**Gene**	**Gene title**	**Statistical Analysis**	***P* Value**	**Difference between Means**	**Difference: Hodges-Lehmann**
**LOX**	Lysyl oxidase	Unpaired t-test with Welch's correction	0.0014	1.06	-
**VEGFA**	Vascular Endothelial Growth Factor A	Unpaired t-test with Welch's correction	0.0002	1	-
**IGFBP3**	Insulin-like growth factor binding protein 3	Unpaired t-test with Welch's correction	0.0001	1.13	
**ADM**	Adrenomedullin	Unpaired t-test with Welch's correction	0.0027	1.2	-
**CAV1**	Caveolin 1, caveolae protein, 22kDa	Unpaired t-test with Welch's correction	0.0005	1.02	-
**CTSB**	Cathepsin B	Unpaired t-test with Welch's correction	0.0121	0.49	-
ALOX5AP	Arachidonate 5-lipoxygenase-activating protein	Unpaired t-test with Welch's correction	0.067	0.59	-
**PLAUR**	Plasminogen activator, urokinase receptor	Mann Whitney test	0.0092	-	0.27
**STC1**	Stanniocalcin 1	Mann Whitney test	<0.0001	-	1.02
LGALS3	Lectin, galactoside-binding, soluble, 3	Mann Whitney test	0.1047	-	0.57
VEGFB	Vascular endothelial growth factor B	Mann Whitney test	0.7186	-	0.03
VEGFC	Vascular endothelial growth factor C	Mann Whitney test	0.5227	-	-0.08
**ANGPTL4**	Angiopoietin-like 4	Mann Whitney test	0.01	-	0.53
**PGK1**	Phosphoglycerate kinase 1	Mann Whitney test	0.0002	-	0.24
**LDHA**	Lactate dehydrogenase A	Mann Whitney test	0.009	-	0.47
SPP1	Secreted phosphoprotein 1	Mann Whitney test	0.1026	-	0.27
**IL6**	Interleukin 6	Mann Whitney test	0.0034	-	0.46
**CA12**	Carbonic anhydrase XII	Mann Whitney test	0.0323	-	0.49

**Note:** Negative values indicate a higher expression in the NTT group.

**Table 1c:** Extracellular matrix signature - Positive values indicate a higher expression in the NOS group.

**Gene**	**Gene Title**	**Statistical Analysis**	***P* Value**	**Difference between Means**	**Difference: Hodges-Lehmann**
GPNMB	Glycoprotein (transmembrane) nmb	Unpaired t-test with Welch's correction	0.3637	0.30	-
**THBS1**	Thrombospondin 1	Unpaired t-test with Welch's correction	0.0592	0.81	-
**COL3A1**	Collagen, type III, alpha 1	Unpaired t-test with Welch's correction	0.0001	1.53	-
**ADAM12**	ADAM metallopeptidase domain 12	Unpaired t-test with Welch's correction	0.0236	0.53	-
**MGP**	Matrix Gla protein	Unpaired t-test with Welch's correction	0.0095	0.97	-
**CDH11**	Cadherin 11, type 2, OB-cadherin (osteoblast)	Unpaired t-test with Welch's correction	0.0004	0.45	-
**CD163**	CD163 molecule	Mann Whitney test	0.0032	-	1.21
LTBR	Lymphotoxin beta receptor (TNFR superfamily, member 3)	Mann Whitney test	0.0934	-	0.17
**CYP1B1**	Cytochrome P450, family 1, subfamily B, polypeptide 1	Mann Whitney test	0.0515	-	0.51
**COL6A3**	Collagen, type VI, alpha 3	Mann Whitney test	0.0005	-	1.12
**IL1R1**	Interleukin 1 receptor, type I	Mann Whitney test	0.0053	-	0.78
MRC1	Mannose receptor, C type 1	Mann Whitney test	0.3912	-	0.22
**FN1**	Fibronectin 1	Mann Whitney test	0.0006	-	0.44

**Note:** Negative values indicate a higher expression in the NTT group.

**Table 1d:** Proliferation signature - Positive values indicate a higher expression in the NOS group.

**Gene**	**Gene Title**	**Statistical Analysis**	***P* Value**	**Difference between Means**	**Difference: Hodges-Lehmann**
RRM2	Ribonucleotide reductase M2	Unpaired t-test with Welch's correction	0.0854	0.48	-
**CENPF**	Centromere protein F, 350/400kDa	Unpaired t-test with Welch's correction	0.0562	0.56	-
TOP2A	Topoisomerase (DNA) II alpha 170kDa	Unpaired t-test with Welch's correction	0.1861	0.46	-
UBE2C	Ubiquitin-conjugating enzyme E2C	Unpaired t-test with Welch's correction	0.0969	0.56	-
BUB1B	BUB1 mitotic checkpoint serine/threonine kinase B	Unpaired t-test with Welch's correction	0.1549	0.37	-
TRIO	Trio Rho guanine nucleotide exchange factor	Unpaired t-test with Welch's correction	0.3904	0.17	-
POLE2	Polymerase (DNA directed), epsilon 2, accessory subunit	Unpaired t-test with Welch's correction	0.2467	0.25	-
CHEK1	Checkpoint kinase 1	Unpaired t-test with Welch's correction	0.2074	0.33	-
CKS2	CDC28 protein kinase regulatory subunit 2	Mann Whitney test	0.3809	-	0.40
**TK1**	Thymidine kinase 1, soluble	Mann Whitney test	0.0121	-	0.24
CENPE	Centromere protein E, 312kDa	Mann Whitney test	0.5137	-	0.14
RAMP1	Receptor (G protein-coupled) activity modifying protein 1	Mann Whitney test	0.8921	-	0.02

**Note:** Negative values indicate a higher expression in the NTT group.

**Table 2a:** Immune signature - Positive values indicate a higher expression in the NOS group.

**Gene**	**Gene Title**	**Statistical Analysis**	***P* Value**	**Difference between Means**	**Difference: Hodges-Lehmann**
***CSF1R***	Colony-stimulating factor 1 receptor	Unpaired t-test with Welch's correction	<0.0001	-0.3764	-0.3883
***FPR1***	Formyl peptide receptor 1	Unpaired t-test with Welch's correction	0.0343	-0.0747	-0.1404
***A2M***	Alpha-2-macroglobulin	Unpaired t-test with Welch's correction	0.008	-0.2314	-0. 1874
*TLR2*	Toll-like receptor 2	Unpaired t-test with Welch's correction	0.3652	-0.0482	-0.082
*FCGR2A*	Fc fragment of IgG, low-affinity IIa, receptor	Unpaired t-test with Welch's correction	0.7983	0.037	-0.0253
***C1R***	Complement component 1, r subcomponent	Unpaired t-test with Welch's correction	0.003	0.4609	0.3464
***C1QB***	Complement component 1, q subcomponent, B chain	Unpaired t-test with Welch's correction	0.0078	-0.2163	-0.2618
*TGFBR2*	Transforming growth factor, beta receptor II	Unpaired t-test with Welch's correction	0.8244	0.0121	-0.0122
*CTSH*	Cathepsin H	Unpaired t-test with Welch's correction	0.1625	-0.2276	-0.1453
***SOCS3***	Suppressor of cytokine signaling 3	Unpaired t-test with Welch's correction	0.0065	0.047	0.0598
***CD44***	CD44 molecule (Indian blood group)	Unpaired t-test with Welch's correction	<0.0001	0.641	0.5409
*TGFB2*	Transforming growth factor, beta 2	Unpaired t-test with Welch's correction	0.6563	-0.0098	-0.0276
***CSF1***	Colony-stimulating factor 1 (macrophage)	Unpaired t-test with Welch's correction	0.0142	0.056	0.0511
*FCGRT*	Fc fragment of IgG, receptor, transporter, alpha	Unpaired t-test with Welch's correction	0.98	-0.0289	-0.0014
***TNFAIP3***	Tumor necrosis factor, alpha-induced protein 3	Mann Whitney test	0.0225	0.2196	0.182
*IL1R1*	Interleukin 1 receptor, type I	Mann Whitney test	0.2795	0.0896	0.0776
***TGFBI***	Transforming growth factor, beta-induced	Mann Whitney test	<0.0001	0.5423	0.5002
*CXCL9*	Chemokine (C-X-C motif) ligand 9	Mann Whitney test	0.9041	0.0339	0.0101
***CD86***	CD86 molecule	Mann Whitney test	0.0014	-0.3116	-0.26
***AIF1***	Allograft Inflammatory Factor 1	Mann Whitney test	0.0004	-0.2181	-0.3514
***C1QA***	Complement component 1, q subcomponent, A chain	Mann Whitney test	0.0206	-0.2185	-0.2421
*CAPG*	Capping protein (actin filament), gelsolin-like	Mann Whitney test	0.6104	-0.0213	0.0423
***SYK***	Spleen tyrosine kinase	Mann Whitney test	0.0171	-0.1642	-0.13
*B2M*	beta-2-microglobulin	Mann Whitney test	0.6008	-0.0351	0.0151
*VCAM*	Vascular cell adhesion molecule 1	Mann Whitney test	0.6445	-0.0132	0.0632

**Note:** Negative values indicate a higher expression in the NTT group.

**Table 2b:** Hypoxia signature - Positive values indicate a higher expression in the NOS group.

**Gene**	**Gene Title**	**Statistical Analysis**	***P* Value**	**Difference between Means**	**Difference: Hodges-Lehmann**
**LOX**	Lysyl oxidase	Unpaired t-test with Welch's correction	0.0053	0.6407	0.4327
**VEGFA**	Vascular Endothelial Growth Factor A	Unpaired t-test with Welch's correction	<0.0001	0.6676	0.6202
**IGFBP3**	Insulin-like growth factor binding protein 3	Unpaired t-test with Welch's correction	0.0014	0.3054	0.4557
**ADM**	Adrenomedullin	Unpaired t-test with Welch's correction	<0.0001	0.6191	0.5806
**CAV1**	Caveolin 1, caveolae protein, 22kDa	Unpaired t-test with Welch's correction	0.0062	0.5768	0.3535
CTSB	Cathepsin B	Unpaired t-test with Welch's correction	0.1221	-0.1003	-0.0902
ALOX5AP	Arachidonate 5-lipoxygenase-activating protein	Unpaired t-test with Welch's correction	0.715	-0.1037	-0.041
**STC1**	Stanniocalcin 1	Mann Whitney test	0.004	0.3213	0.2587
**LGALS3**	Lectin, galactoside-binding, soluble, 3	Mann Whitney test	<0.0001	0.5058	0.3981
VEGFB	Vascular endothelial growth factor B	Mann Whitney test	0.8917	0.0467	0.0053
VEGFC	Vascular endothelial growth factor C	Mann Whitney test	0.2576	0.0411	0.0482
**ANGPTL4**	Angiopoietin-like 4	Mann Whitney test	0.0281	0.2387	0.2794
**PGK1**	Phosphoglycerate kinase 1	Mann Whitney test	<0.0001	0.4296	0.4414
**LDHA**	Lactate dehydrogenase A	Mann Whitney test	<0.0001	0.1893	0.2047
**SPP1**	Secreted phosphoprotein 1	Mann Whitney test	<0.0001	-0.2274	-0.2162
IL6	Interleukin 6	Mann Whitney test	0.1837	0.1982	0.0966
**CA12**	Carbonic anhydrase XII	Mann Whitney test	<0.0001	0.8185	0.6013

**Note:** Negative values indicate a higher expression in the NTT group.

**Table 2c:** Extracellular matrix signature - Positive values indicate a higher expression in the NOS group.

**Gene**	**Gene Title**	**Statistical Analysis**	***P* Value**	**Difference between Means**	**Difference: Hodges-Lehmann**
GPNMB	Glycoprotein (transmembrane) nmb	Unpaired t-test with Welch's correction	0.342	-0.152	-0.1178
**THBS1**	Thrombospondin 1	Unpaired t-test with Welch's correction	0.0006	0.2859	0.2642
**COL3A1**	Collagen, type III, alpha 1	Unpaired t-test with Welch's correction	0.0156	0.3926	0.3979
**ADAM12**	ADAM metallopeptidase domain 12	Unpaired t-test with Welch's correction	0.0004	0.2088	0.1789
MGP	Matrix Gla protein	Unpaired t-test with Welch's correction	0.6537	0.1603	0.0577
**CDH11**	Cadherin 11, type 2, OB-cadherin (osteoblast)	Unpaired t-test with Welch's correction	0.003	0.2689	0.1974
CD163	CD163 molecule	Mann Whitney test	0.1619	0.3725	0.2143
LTBR	Lymphotoxin beta receptor (TNFR superfamily, member 3)	Mann Whitney test	0.5628	0.0631	0.0308
CYP1B1	Cytochrome P450, family 1, subfamily B, polypeptide 1	Mann Whitney test	0.7444	0.0252	0.0488
**COL6A3**	Collagen, type VI, alpha 3	Mann Whitney test	0.0007	0.6655	0.6003
IL1R1	Interleukin 1 receptor, type I	Mann Whitney test	0.2795	0.0896	0.0776
MRC1	Mannose receptor, C type 1	Mann Whitney test	0.2258	0.2938	0.1614
FN1	Fibronectin 1	Mann Whitney test	0.5281	0.0505	0.0515

**Note:** Negative values indicate a higher expression in the NTT group.

**Table 2d:** Proliferation signature - Positive values indicate a higher expression in the NOS group.

**Gene**	**Gene Title**	**Statistical analysis**	***P* Value**	**Difference between Means**	**Difference: Hodges-Lehmann**
**RRM2**	Ribonucleotide reductase M2	Unpaired t-test with Welch's correction	<0.0001	0.3847	0.3711
CENPF	Centromere protein F, 350/400kDa	Unpaired t-test with Welch's correction	0.067	0.2515	0.1806
**TOP2A**	Topoisomerase (DNA) II alpha 170kDa	Unpaired t-test with Welch's correction	0.0195	0.1446	0.2749
**UBE2C**	Ubiquitin-conjugating enzyme E2C	Unpaired t-test with Welch's correction	<0.0001	0.4146	0.4502
**TRIO**	Trio Rho guanine nucleotide exchange factor	Unpaired t-test with Welch's correction	0.0132	0.2371	0.2067
**POLE2**	Polymerase (DNA directed), epsilon 2, accessory subunit	Unpaired t-test with Welch's correction	<0.0001	0.3863	0.3926
**CHEK1**	Checkpoint kinase 1	Unpaired t-test with Welch's correction	<0.0001	0.3553	0.358
**CKS2**	CDC28 protein kinase regulatory subunit 2	Mann Whitney test	0.0037	0.2519	0.2745
**TK1**	Thymidine kinase 1, soluble	Mann Whitney test	<0.0001	0.243	0.2593

**Note:** Negative values indicate a higher expression in the NTT group.

**Table 3:** Positive values indicate a higher expression in the NOS group.

**Gene**	**Gene Title**	**Statistical Analysis**	***P* Value**	**Difference between Means**	**Difference: Hodges-Lehmann**
**SCD**	Stearoyl-CoA Desaturase (delta-9-desaturase)	Unpaired t-test with Welch's correction	0.01	-0.45	-
**SIK3**	SIK family kinase 3	Unpaired t-test with Welch's correction	0.0006	-0.77	-
**NEGR1**	Neuronal growth regulator 1	Unpaired t-test with Welch's correction	0.0003	-1.02	-
**GABRB3**	Gamma-aminobutyric acid (GABA) A receptor, beta 3	Unpaired t-test with Welch's correction	<0.0001	-1.57	-
**GRID1**	Glutamate receptor, ionotropic, delta 1	Unpaired t-test with Welch's correction	0.0007	-0.36	-
**SCN2A**	Sodium channel, voltage-gated, type II, α subunit	Unpaired t-test with Welch's correction	<0.0001	-0.91	-
**RDH10**	Retinol dehydrogenase 10 (all-trans)	Unpaired t-test with Welch's correction	<0.0001	1.46	-
**BRINP1**	Bone morphogenetic protein/retinoic acid-inducible neural-specific 1	Unpaired t-test with Welch's correction	<0.0001	-1.27	-
**H3F3A**	H3 histone, family 3A	Unpaired t-test with Welch's correction	0.0001	0.38	-
**GABRB2**	Gamma-aminobutyric acid (GABA) A receptor, beta 2	Unpaired t-test with Welch's correction	0.0002	-1.22	-
**FA2H**	Fatty acid 2-hydroxylase	Unpaired t-test with Welch's correction	<0.0001	-1.48	-
**MOG**	Myelin oligodendrocyte glycoprotein	Unpaired t-test with Welch's correction	0.0003	-1.54	-
**NEGR1**	Neuronal growth regulator 1	Unpaired t-test with Welch's correction	0.0003	-1.02	-
**ANK3**	Ankyrin 3, node of Ranvier (ankyrin G)	Mann Whitney test	<0.0001	-	-1.05
**MTURN**	Maturin, neural progenitor differentiation regulator homolog (Xenopus)	Mann Whitney test	0.0018	-	-0.61
FAR1	Fatty acyl CoA reductase 1	Mann Whitney test	0.2583	-	-0.26
**MOBP**	Myelin-associated oligodendrocyte basic protein	Mann Whitney test	<0.0001	-	-1.68
IGF1R	Insulin-like growth factor 1 receptor	Mann Whitney test	0.7825	-	-0.07
**MBP**	Myelin basic protein	Mann Whitney test	0.0008	-	-1.70
**GRIA2**	Glutamate receptor, ionotropic, AMPA 2	Mann Whitney test	0.0021	-	-0.83
**KCNK10**	Potassium channel, subfamily K, member 10	Mann Whitney test	0.0029	-	-0.35

**Note:** Negative values indicate a higher expression in the NTT group.

**Table 4:** Positive values indicate a higher expression in the NOS group.

**Gene**	**Gene Title**	**Statistical Analysis**	***P* Value**	**Difference between Means**	**Difference: Hodges-Lehmann**
SCD	Stearoyl-CoA Desaturase (delta-9-desaturase)	Unpaired t-test with Welch's correction	0.928	0.0388	-0.0053
**GABRB3**	Gamma-aminobutyric acid (GABA) A receptor, beta 3	Unpaired t-test with Welch's correction	<0.0001	-0.1329	-0.1346
**SCN2A**	Sodium channel, voltage-gated, type II, α subunit	Unpaired t-test with Welch's correction	<0.0001	-0.5647	-0.6182
GABRB2	Gamma-aminobutyric acid (GABA) A receptor, beta 2	Unpaired t-test with Welch's correction	0.6412	0.0183	0.0105
**FA2H**	Fatty acid 2-hydroxylase	Unpaired t-test with Welch's correction	<0.0001	-1.436	-1.187
**MOG**	Myelin oligodendrocyte glycoprotein	Unpaired t-test with Welch's correction	<0.0001	-2.588	-2.009
**ANK3**	Ankyrin 3, node of Ranvier (ankyrin G)	Mann Whitney test	<0.0001	-0.6594	-0.7357
**MOBP**	Myelin-associated oligodendrocyte basic protein	Mann Whitney test	<0.0001	-2.379	-1.909
IGF1R	Insulin-like growth factor 1 receptor	Mann Whitney test	0.0627	0.0763	0.1273
**MBP**	Myelin basic protein	Mann Whitney test	<0.0001	-1.968	-2.061
**GRIA2**	Glutamate receptor, ionotropic, AMPA 2	Mann Whitney test	<0.0001	-0.7195	-0.846
**KCNK10**	Potassium channel, subfamily K, member 10	Mann Whitney test	0.0002	-0.2097	-0.2085

**Note:** Negative values indicate a higher expression in the NTT group.

## Data Availability

Not applicable.

## LIST OF ABBREVIATIONS

ECM: Extracellular Matrix
GBM: Glioblastoma Multiforme
GSCs: Glioma Stem Cells
NAMs: Negative Allosteric Modulators
NFkB: Nuclear Factor-kB
PI3K: Phosphatidylinositol-3-kinase
RMA: Robust Multi-array Average
